# Immunoinformatics approach to epitope-based vaccine design against the SARS-CoV-2 in Bangladeshi patients

**DOI:** 10.1186/s43141-022-00410-8

**Published:** 2022-09-20

**Authors:** Shahina Akter, Muhammad Shahab, Md. Murshed Hasan Sarkar, Chandni Hayat, Tanjina Akhtar Banu, Barna Goswami, Iffat Jahan, Eshrar Osman, Mohammad Samir Uzzaman, Md Ahashan Habib, Aftab Ali Shaikh, Md. Salim Khan

**Affiliations:** 1 Bangladesh Council of Scientific & Industrial Research (BCSIR), Dhaka, Bangladesh; 2 State Key Laboratories of Chemical Resources Engineering, Beijing University of Chemical Technology, Beijing, 100029 China; 3 Department of Biochemistry, Computational Medicinal Chemistry Laboratory, UCSS, Abdul Wali Khan University, Mardan, Pakistan; 4 SciTech Consulting and Solutions, Dhaka, Bangladesh

**Keywords:** SARS-CoV-2, Epitope, Vaccine, B cell, T cell, Immunoinformatics

## Abstract

**Background:**

Severe acute respiratory syndrome coronavirus 2 (SARS-CoV-2) is the causative agent of the ongoing coronavirus disease 2019 (COVID-19) pandemic which has brought a great challenge to public health. After the first emergence of novel coronavirus SARS-CoV-2 in the city of Wuhan, China, in December 2019. As of March 2020, SARS-CoV-2 was first reported in Bangladesh and since then the country has experienced a steady rise in infections, resulting in 13,355,191 cases and 29,024 deaths as of 27 February 2022. Bioinformatics techniques are used to predict B cell and T cell epitopes from the new SARS-CoV-2 spike glycoprotein in order to build a unique multiple epitope vaccine. The immunogenicity, antigenicity scores, and toxicity of these epitopes were evaluated and chosen based on their capacity to elicit an immune response.

**Result:**

The best multi-epitope of the possible immunogenic property was created by combining epitopes. EAAAK, AAY, and GPGPG linkers were used to connect the epitopes. In several computer-based immune response analyses, this vaccine design was found to be efficient, as well as having high population coverage.

**Conclusion:**

This research is entirely reliant on the development of epitope-based vaccines, and these *in silico* findings would represent a major step forward in the development of a vaccine that might eradicate SARS-CoV-2 in Bangladeshi patients.

## Introduction

SARS-CoV-2 is the cause of the current COVID-19 pandemic, which has brought the serious threat to public health [1]. In December 2019, the new coronavirus SARS-CoV-2 was discovered in Wuhan, China, causing a worldwide disaster known as COVID-19, which has spread to 217 countries and territories [2]. In March 2020, SARS-CoV-2 illnesses first reported in Bangladesh and the country has seen a steady increase in infections with 13,355,191 cases and 29,024 deaths as of February 27, 2022 (https://corona.gov.bd/?gclid).

Globally, the World Health Organization recorded 430,257564 confirmed cases and 5,922049 fatalities, prompting major health concerns [3]. In this case, avoiding and controlling this infectious illness is crucial. Despite the fact that a global vaccination program is on the spot, novel lineages comprising a cluster of mutations in the receptor-binding domain and immunodominant areas of the N terminal domain of spike glycoprotein have developed, resulting in immunological escape variants with higher transmissibility and infectivity [4]. According to studies, these variants can possibly lead to resistance against protective antibodies induced by natural infection or immunization. In certain cases, it shows total immunological escape in convalescent serum samples, raising concerns about the effectiveness and efficacy of currently available vaccinations [5]. On 9th of February 2021, Bangladesh launched a national mass vaccination campaign among people by Oxford AstraZeneca (https://www.imago-images.com/st/0112541387). As of March 2022, more than 140 million doses of currently approved vaccines had been administered in Bangladesh. Bangladesh observed the second wave of COVID-19 in March 2021, employing the UK variant as well as the Beta, V2 variant from South Africa, during a vaccination program. Furthermore, the third wave of COVID-19, caused by Delta variant, arrived in mid-May 2021 and in Bangladesh, it turned out to be the most lethal, resulting in a huge increase in COVID-19 cases and deaths domestically. The Omicron (B.1.1.529) a new variant was identified in November from South Africa and quickly spread to over 57 countries throughout the world. According to recent research, the development of complicated mutations in the spike glycoprotein may enable this variant to elude the protective immune response induced by vaccination.

On January 12, 2020, Chinese scientists disclosed and reported the SARS-CoV-2 genome sequence, which was then uploaded to the GenBank database [6]. As the total genome sequencing of the Corona virus is done, the complexity has been studied by scientists. The knowledge of SARS-CoV-2 contagion and latency is currently poor, raising concerns about viral persistence. Vaccination is the most cost-effective and efficient method of preventing viral infection. There are currently no specific antiviral therapeutics available [7]. The identification and production of pathogen-specific protective immunogens, especially for newly emerging viruses, is a key problem in vaccine design.

Researchers have proposed a number of ways for developing SARS-CoV-2 vaccines [8]. The standard vaccine design approach on the basis of lab studies, isolating, inactivating, and injecting the virus is a time-consuming technique, and does not fulfil the urgent needs of an outbreak. To accelerate the development of vaccines, researchers subsequently focused on methods based on immunoinformatics to develop multi-epitope vaccines [9]. Different virus protein segments rich in overlapping epitopes are used to make multi-epitope vaccinations. They contain the virus essential components for provoking an immunological response, either cellular or humoral, as well as undesired components that can cause side effects [10]. In silico analysis can anticipate the virus characteristics as well as the pathogen epitopes, which can greatly speed up vaccine development process [11, 12]. The main objective of this work is the SARS-CoV-2 spike protein sequence, which will be used to develop a multi-epitope vaccine. Bioinformatics and immunoinformatics tools were utilized to anticipate T cell epitopes inside the nucleocapsid protein of SARS-CoV-2 and B cell epitopes on the spike protein. The new epitope prediction and information could contribute to the development of a promising SARS-CoV-2 vaccine against escape variants.

## Methods

### Sample collection and screening for SARS-CoV-2

Nasopharyngeal specimens were collected from suspected COVID-19 patients in Bangladesh. Specimens were kept at 4 °C, before and during transport when sampled and processed on the day of collection or stored at – 20 °C. The specimens were transported on ice and processed within 24 h of collection.

Cycle threshold (Ct) values were used to select nasopharyngeal swab samples for RNA extraction. SARS-CoV-2 has predominantly been detected using the SANSURE qRT-PCR kit (PCR-Fluorescence Probing, Sansure Biotech Inc.) according to the manufacturer’s instructions. The qRT-PCR was performed for *ORF1ab* and *N* genes of SARS-CoV-2.

### Extraction of viral nucleic acid, PCR amplification, and mRNA sequencing

Whole-genome sequencing of SARS-CoV-2 isolates were conducted by the Genomic Research Laboratory, BCSIR, Dhaka, Bangladesh using NextSeq 550. In this case, ReliaPrep^TM^ Viral Total Nucleic Acid Purification Kit was used (catalog no. AX4820); Illumina RNA Prep with Enrichment, (L) Tagmentation protocol were followed with step-by-step instructions to prepare and enrich libraries (Illumina, catalogue no. 1000000124435). A paired-end (2 × 74 bp) read was generated from each sample after tagmentation and amplification. Based on default parameters, Basespace Dragen RNA Pathogen Detection programme (version 3.5.14) was used to construct SARS-CoV-2 genome sequence reads.

### SARS-CoV-2 genome assembly and quality control

SARS-CoV-2 genome readings were found in qRT-PCR positive samples after sequencing. The sequencing method generates 1.2 million reads per sample. With EzCOVID19 (https://www.ezbiocloud.net/tools/ sc2) of EZBioCloud, the samples are subjected to SARS-CoV-2 consensus generation. SARS-CoV-2 genome coverage averaged 2×.

### Sequence retrieval

Whole-genome sequence of 15 isolates of SARS-CoV-2 were done using NextSeq 550. SARS-CoV-2 spike glycoprotein sequence was extracted from the FASTA file after alignment of 15 SARS-CoV-2 isolates with a reference sequence (Wuhan) using Mega-X. From all the data the spike glycoprotein of BCSIR-NILMRC_355 was selected for vaccine design as the data quality of this isolates was good. The PDB file of the particular protein was built using Swiss-model (https://swissmodel.expasy.org/interactive). Protein structure identification and analyses were reported through Expasy (https://web.expasy.org/protparam/) [13]. Secondary structure of protein was predicated trough PSIPRED (http://bioinf.cs.ucl.ac.uk/psipred/) [14]. Disulfide bond was predicted through DiANNA (http://clavius.bc.edu/~clotelab/DiANNA/) [15].

### Prioritization of vaccine protein

To determine the antigenic behaviors of the vaccine protein, the sequence was first subjected to online server VaxiJen (http://www.ddg-pharmfac.net/vaxijen/VaxiJen/VaxiJen.html) with the threshold 4.0 [16]. while allergenicity was checked through AllerTop v.2 ( https; /www.ddg.pharmfac.net/AllerTop). The trRosetta [16] and I-TASSER tool were used to obtain the 3D structure of proteins. TMHMM, an online tool, was used to predict transmembrane topology (https://services.healthtech.dtu.dk/service.php?TMHMM-2.0).

### Prediction of B cell and T cell epitopes

Selecting effective B cell epitopes requires surface accessibility. Thus, the Emini surface accessibility tool was used to analyses. IEDB Resource (http://tool.iedb.org/main/) [17] was used to predict the B cell and T cell epitopes and the binding scores were calculated for T cell epitope related to MHC-I and MHC-II. The threshold value was kept at 0.5 and IC_50_ scores were assigned by the online server. A threshold score of more than 0.5 was taken, good epitope candidate. IC_50_ and binding affinity are inversely proportional to each other. Mean if IC_50_ value is small, epitope binding affinity to MHC-II will be high. IC_50_ values < 10 nM < 100 nM < 1000 nM means high, intermediate and low binding affinity with MHC-II. MHC-II and binding affinity between epitope are inversely proportional to percentile rank [18].

### Antigenicity and allergenicity prediction of B cell and T cell epitopes

To check the antigenic and allergic behavior of B cells and T cells epitopes, online servers VaxiJen v2.0 [19] and AllerTop [20] were used. The epitope with higher value than the reference and non-allergic was chosen for further research.

### Population coverage

Using online IEDB Analysis Resource, MHC-I and MHC-II epitopes were used to predict population coverage (tools.iedb.org/population/). The recovery score of more than 60 was considered as a good population coverage [21]. A vaccine construct was designed using B cell and T cell epitopes with high binding affinity, non-allergenicity, and antigenicity. Based on earlier works [21, 22], the selected epitopes were linked together using linkers, AAY, and GPGPG, and were used to connect all MHC-I and MHC-II epitopes, respectively. The EAAAK linker was used to bind the adjuvant to the N-terminal of the vaccine construct [23].

### Secondary and tertiary structure prediction and validation

PSIPRED 3.3 online server (http://bioinf.cs.ucl.ac.uk/psipred/) and TrRosetta web server were used to generate the secondary and tertiary structures of the vaccine construct. The 3D structure of the vaccine construct was generated after analysis of the domain with 3D structure. Galaxy Refine (https://galaxy.seoklab.org/cgi-bin/submit.cgi?type=REFINE) was used to validate the vaccine. For further verification by ProSA-Web Ramachandran Plot, and PROCHECK (https://saves.mbi.ucla.edu/) [24] online servers were used.

### Molecular docking of vaccine construct

The three-dimensional structure of TLR-4 with (PDB ID: 4G8A) was downloaded from PDB. The structure was first check for missing residues and breaks. The water molecules were removed. The MOE software were used for the correct protonation. Then the structure was save in TLR-4.pdb format. The vaccine sequence was docked with the human Toll-like receptor 4 through Cluspro (https://cluspro.bu.edu) most extensively used docking server, which is based on six energy functions and depends on the type of protein. Cluspro predicts 10 docking results in about 5 h based on densely packed low-energy clusters for each docking parameter. This procedure illustrates how to use various choices such as creating extra restrictions files, selecting various energy parameters, and analyzing the outcomes. PDBsum were used to validate the vaccine and TLR-4 graphical representations.

### Codon optimization and MD simulation

The JCat tool was used to optimize codons and reverse the vaccination sequence [25]. The JCat program is also used to guarantee that the vaccination sequence is expressed in a vector with a high level of expression. Three extra parameters were chosen in this tool, including Rho-independent transcription termination, restriction enzyme cleavage sites, and bacterial ribosome binding sites. JCat determines the CAI score and GC content of the vaccine sequence.

**Fig. 1:**
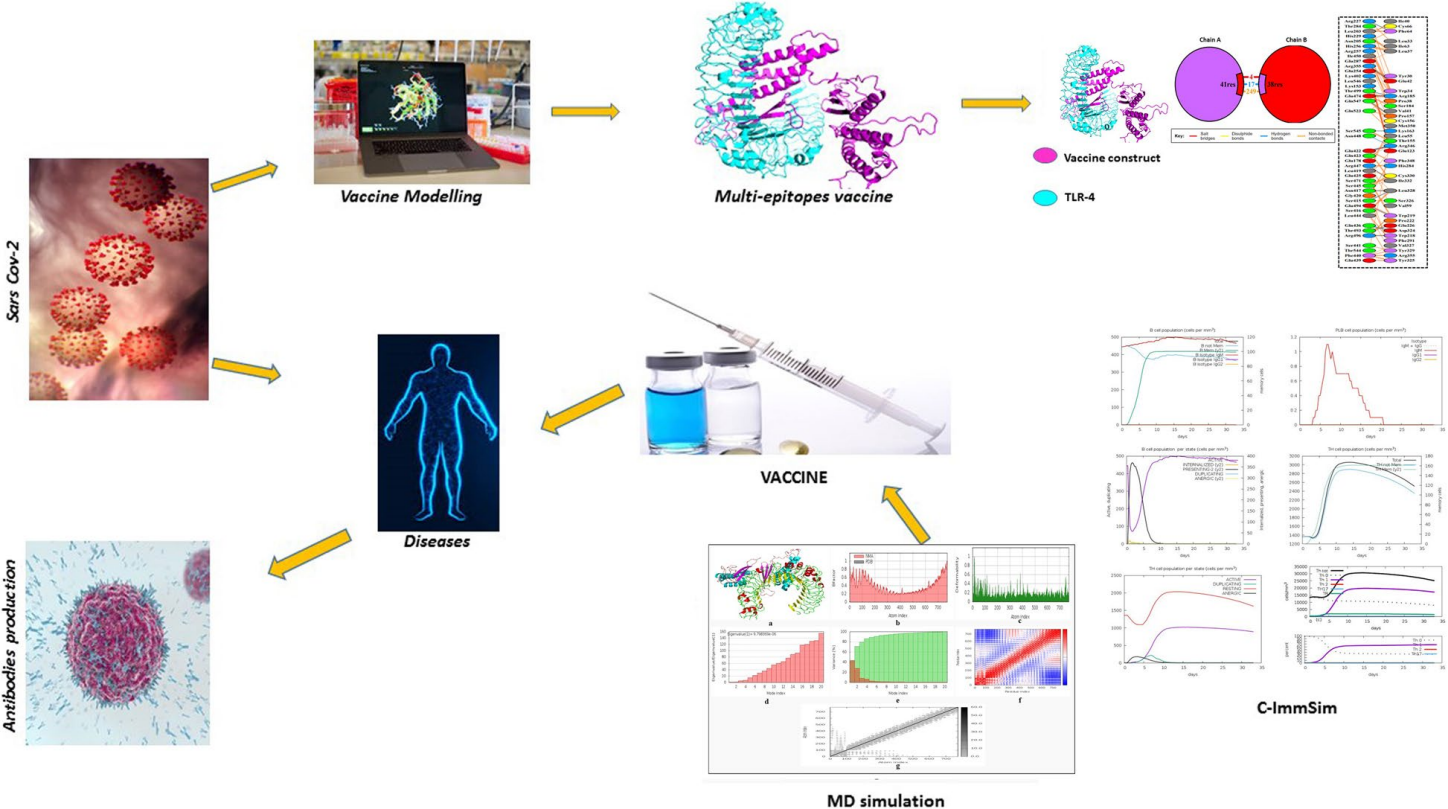
Experimental diagram for epitope-based vaccine design

iMODS, an open-source modelling, for molecular dynamics simulation and image processing applications, was utilized for 3D reconstruction and modelling of the TLR-4-vaccine complex. iMODS, an open-source modelling, tool was used for MD simulation and image processing applications, this server provides a user-friendly interface for internal NMA. Users can perform NMA or simulate possible trajectories between two conformations and view the results in 3D, even for enormous macromolecules. The overall experimental diagram for epitope-based vaccine design are given in Fig. 1.

## Results

### Protein structure prediction

**Fig. 2:**
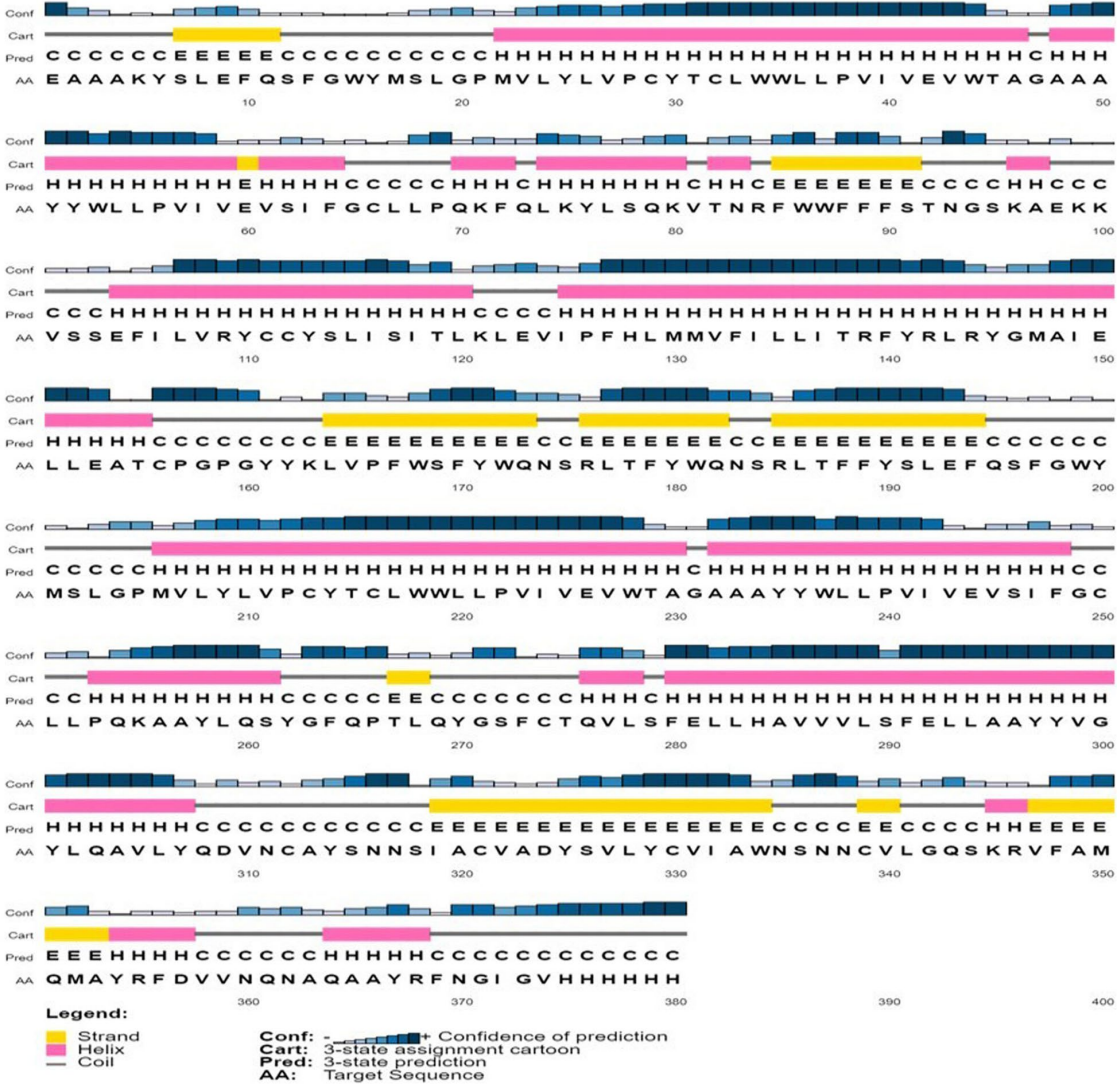
Protein secondary structure prediction: H (helix), E (strands), and C (coils)

SARS-CoV-2 spike glycoprotein has 12.4% β-strands, 46.9% α-helices, and 40.7% coil structures, according to secondary structure prediction using PSIPRED and 3D structure prediction using I-TASSER [26] (Fig. 2). Furthermore, the DiANNA1.1 program [27] estimated 15 disulfide bonds with the assign scores presented in Table 1. TMHMM, an online tool, was used to predict transmembrane topology.

**Table 1:** Disulfide bond prediction calculated by DIANA1.1.S-S bond scores

Cysteine sequence position	Distance (Å)	Bond	Score

6–16	10	PKFTKCRSPER-RETFSCHWTLG	0.01456
6–42	36	PKFTKCRSPER-QEWKECPDYVS	0.01107
6–53	47	PKFTKCRSPER-AGENSCYFNSS	0.01238
6–67	61	PKFTKCRSPER-IWIPYCIKLTS	0.01052
6–81	75	PKFTKCRSPER-TVDEKCFSVDE	0.013
16–42	26	RETFSCHWTLG-QEWKECPDYVS	0.01041
16–53	37	RETFSCHWTLG-AGENSCYFNSS	0.01385
16–67	51	RETFSCHWTLG-IWIPYCIKLTS	0.01045
16–81	65	RETFSCHWTLG-TVDEKCFSVDE	0.01069
42–53	11	QEWKECPDYVS-AGENSCYFNSS	0.99779
42–67	25	QEWKECPDYVS-IWIPYCIKLTS	0.01037
42–81	39	QEWKECPDYVS-TVDEKCFSVDE	0.01063
53–67	14	AGENSCYFNSS-IWIPYCIKLTS	0.0104
53–81	28	AGENSCYFNSS-TVDEKCFSVDE	0.01213
67–81	14	IWIPYCIKLTS-TVDEKCFSVDE	0.90847

Surface exposed residues were detected at position 1–14, 69–114, 222–230, and 305–318, whereas residues from 38 to 48, 138–198, 254–281, and 342–380 were found inside the transmembrane region. The core area of the SARS CoV-2 spike glycoprotein was determined to have residues from positions 15–37, 49–68, 115–137,199–221, and 319–341.

### B cell epitopes recognition

B cell epitopes are crucial for viral infection resistance. Potential B cell epitopes have changed characteristics that instruct B cells to recognize and activate a wide range of immune responses directed to specific viral infections. A total of ten linear epitopes were calculated with a threshold score of 0.500 through IEBD analysis tool. For linear epitope maximum score was 0.698 and the minimum score was 0.191. However, average prediction score was 0.476. Allergenic and poisonous epitopes were eliminated, and only antigenic and non-allergic epitopes were chosen. On average, 10 out of 25 B cell epitopes were considered effective. (RSYLTPGDSSSGFTVEYQTSNFRVQPSNKKFLPFQTLVNNSYECDIPIQLGQSKRVDFCEDR) that can evoke B-lymphocytes. After compensating of data, sequences of peptides from 350 to 440 residues boost the immune responses Table 2. The Kolaskar and Tongaonkar approach was used to test the antigenicity of experimentally predicted epitopes [28]. The antigenicity analysis showed that the minimum antigenicity value was 0.05and the maximum value was 1.713. However, the average value was observed at 1.043. The threshold value was adjusted to 0.4 and values more than 0.4 were categorized as candidates of antigenic factors and used for further analysis. These epitopes potentially activate B cells are shown in Table 3.

**Table 2:** IEDB analysis resource predicts a list of Bepipred linear epitopes

No.	Start	End	Peptide	Length
1	6	17	RSYLTPGDSSSG	12
2	66	69	FTVE	4
3	73	82	YQTSNFRVQP	10
4	315	323	SNKKFLPFQ	9
5	341	342	TL	2
6	416	426	VNNSYECDIPI	11
7	717	717	Q	1
8	794	802	LGQSKRVDFC	10
9	942	942	E	1
10	944	945	DR	2

**Table 3:** Antigenicity prediction using the Kolaskar and Tongaonkar technique

Start	End	Peptide	Length
223	233	YSLEFQSFGW	10
314	322	YMSLGPMVL	9
812	820	YLVPCYTCL	9
301	310	YYKLVPFWSF	10
962	970	YWQNSRLTF	9
590	598	YSLEFQSFGW	10
148	156	YLVPCYTCL	9
92	101	WWLLPVIVEV	10
448	456	WTAGAAAYY	9
193	201	WLLPVIVEV	9
130	139	SIFGCLLPQK	10

Selecting effective B cell epitopes requires surface accessibility. Thus, the Emini surface accessibility tool was used to analyses. After detecting the default threshold value of 1.000, 13 epitopes were selected. The area with the most accessibility was 89–95 residues, with an average observed value of 1.00. Although the least value was 0.078 and the greatest value was 5.199, it was discovered that the minimum value was 0.078, maximum value was 5.199. Table 4 shows the results of conservancy analysis on B cell epitopes using the IEDB program. In all, 5 epitopes were chosen for conservancy investigation and will be used in vaccine development. These epitopes are extremely conserved, with 100% coverage and identity.

**Table 4:** Prediction of Emini surface accessibility for the accessible region

Start	End	Peptide	Length
113	121	LQSYGFQPT	9
220	228	LQYGSFCTQ	9
284	292	VLSFELLHA	9
302	310	VVVLSFELL	9
562	570	AAYYVGYLQ	9
590	598	AVLYQDVNC	9
48	56	AYSNNSIAI	9
92	100	CVADYSVLY	9
412	420	CVIAWNSNN	9
890	898	CVLGQSKRV	9
131	139	FAMQMAYRF	9
902	910	DVVNQNAQA	9
966	974	AYRFNGIGV	9

### T cell epitope prediction

#### Prediction of MHC-1 epitope

We employed SMM to study MHC HLA alleles in *Homo sapiens* as the MHC source. This tool can compute epitope HLA binding affinity using the IC_50_ nM unit. A lesser IC_50_ suggests greater affinity for MHC-I molecules. In order to maximize affinity for MHC-1 alleles, the total number of epitopes was designed to be fewer than 100. On the basis of MHC-1 allele interactions, 60 epitopes were chosen among 950. The antigenicity, allergenicity, and toxicity of 60 epitopes were assessed. The antigenic scores of toxic and allergic epitopes were ruled out. The MHC-1 epitopes were finalized. They bind to alleles HLA-B*58:01, HLA-B*57:01, HLA-A*23:01, and HLA-A*24:02. YSLEFQSFGW has an antigenic score of 1.713 (Table 3).

#### Prediction of MHC-II epitope

MHC-II alleles interacted with 550 conserved predicted epitopes with IC_50_ under 60. Thirty epitopes were selected among 550 that interacted with 5 MHC-II alleles. Thirteen epitopes were selected for further study on the basis of their allergenicity, toxicity, and antigenicity. The epitope CVADYSVLY are FAMQMAYRF, and AYSNNSIAI were considered the top binder, with alleles HLA-DRB1*03:01, HLA-DRB1*04:01, HLA-DRB1*07:01, HLA-DRB1*13:02, HLA-DRB3*02:02) (Table 4).

### Multi-epitope subunit vaccine construction

We chose 10 B cell epitopes, and T Cell epitope (11 MHC-I and 13 MHC-II) for the vaccination chimaeras. The vaccine included 50S ribosomal protein L7/L12 as an adjuvant. The adjuvant and the B cell epitope was linked by EAAAK linker. B cell and MHC-I epitopes were linked by GPGPG linkers and MHC-I and MHC-II epitopes was linked through AAY linkers. The vaccine was made smaller by combining B cell, CTL, and HLT epitopes. The vaccine sequence has a 6x His tag for protein identification and purification. The resulting vaccine construct sequence has a molecular weight of 50689.18 kDa and 309 a.a sequence. The Vaccine build is shown in the diagram below.

>vaccine protein

EAAAKYSLEFQSFGWYMSLGPMVLYLVPCYTCLWWLLPVIVEVWTAGAAAYYWLLPVIVEVSIFGCLLPQKFQLKYLSQKVTNRFWWFFFSTNGSKAEKKVSSEFILVRYCCYSLISITLKLEVIPFHLMMVFILLITRFYRLRYGMAIELLEATCPGPGYYKLVPFWSFYWQNSRLTFYWQNSRLTFFYSLEFQSFGWYMSLGPMVLYLVPCYTCLWWLLPVIVEVWTAGAAAYYWLLPVIVEVSIFGCLLPQKAAYLQSYGFQPTLQYGSFCTQVLSFELLHAVVVLSFELLAAYYVGYLQAVLYQDVNCAYSNNSIACVADYSVLYCVIAWNSNNCVLGQSKRVFAMQMAYRFDVVNQNAQAAYRFNGIGVHHHHHH.

### Physicochemical properties of the vaccine

Protparam (Expasy) (https://web.expasy.org/protparam/) was used to calculate the physical and chemical parameters of the vaccine, which consisted of 380 residues, and a molecular weight of 50689.18 kDa. The theoretical isoelectric point (PI) value is 8.96 which indicates that the protein is positively charged, as is the case with isoelectric points over 7.0. An instability index (II) of 42.46 determined by Protparam classified our protein to be stable. The aliphatic index is 73.10 which is thermo-stable across a wide temperature range. The aliphatic index is 73.10, indicating that it is thermo-stable over a wide temperature range. At 0.007, the grand average of hydropathcity (GRAVY) was computed using the chemical formula C2283H3452N620O664S15.

### Epitope population coverage and conservancy analysis

The MHC-I allele was found to be present in 91.1% of the world’s population, followed by South Asia including Bangladesh (91.18%), Northeast Asia (91.85%), South Asia (95.13%), Europe (95.13%), and North America (88.65%). The lowest population was found in Central America (7.76%). South Asia has the highest percentage of MHC-I alleles in the population (91%), followed by Europe. Central Africa was found to have the lowest population density (51.46 %) [22].

Four epitopes (LQSYGFQPT, LQYGSFCTQ, VLSFELLHA, and VVVLSFELL) are crucial for the majority of interactions in MHC-II alleles describe a considerable coverage in contrast to the whole world population. For LQSYGFQPT, the proportion of concentrated population coverage in the world was anticipated at 69.75%. In the instance of MHC-I, five epitopes (SIFGCLLPQK, WLLPVIVEV, WWLLPVIVEV, YLVPCYTCL, and YMSLGPMVL) were predicted to interact with common MH Class-I alleles, resulting in a massive worldwide coverage. The population coverage data for the abundant binders to MHC-I and MHC-II alleles reveal 97.55 and 98.65% coverage, respectively [29].

### Multi-epitope 3D structure and structural validation of vaccine

**Fig. 3:**
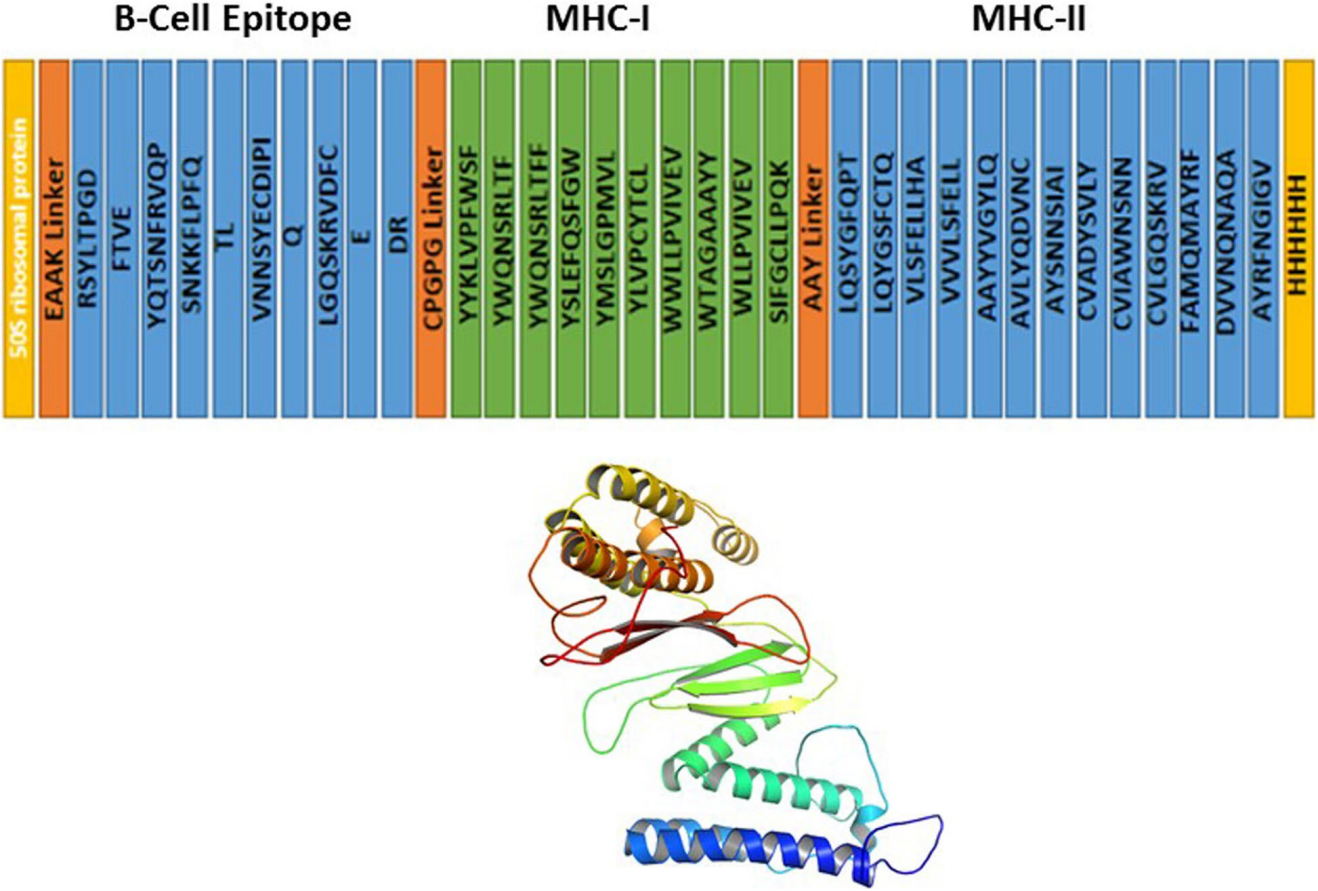
Graphical and 3D view of vaccine construction. 50 s ribosomal protein, EAAAL Linker, CPGPG Linker, and AAY Linker are shown

**Fig. 4:**
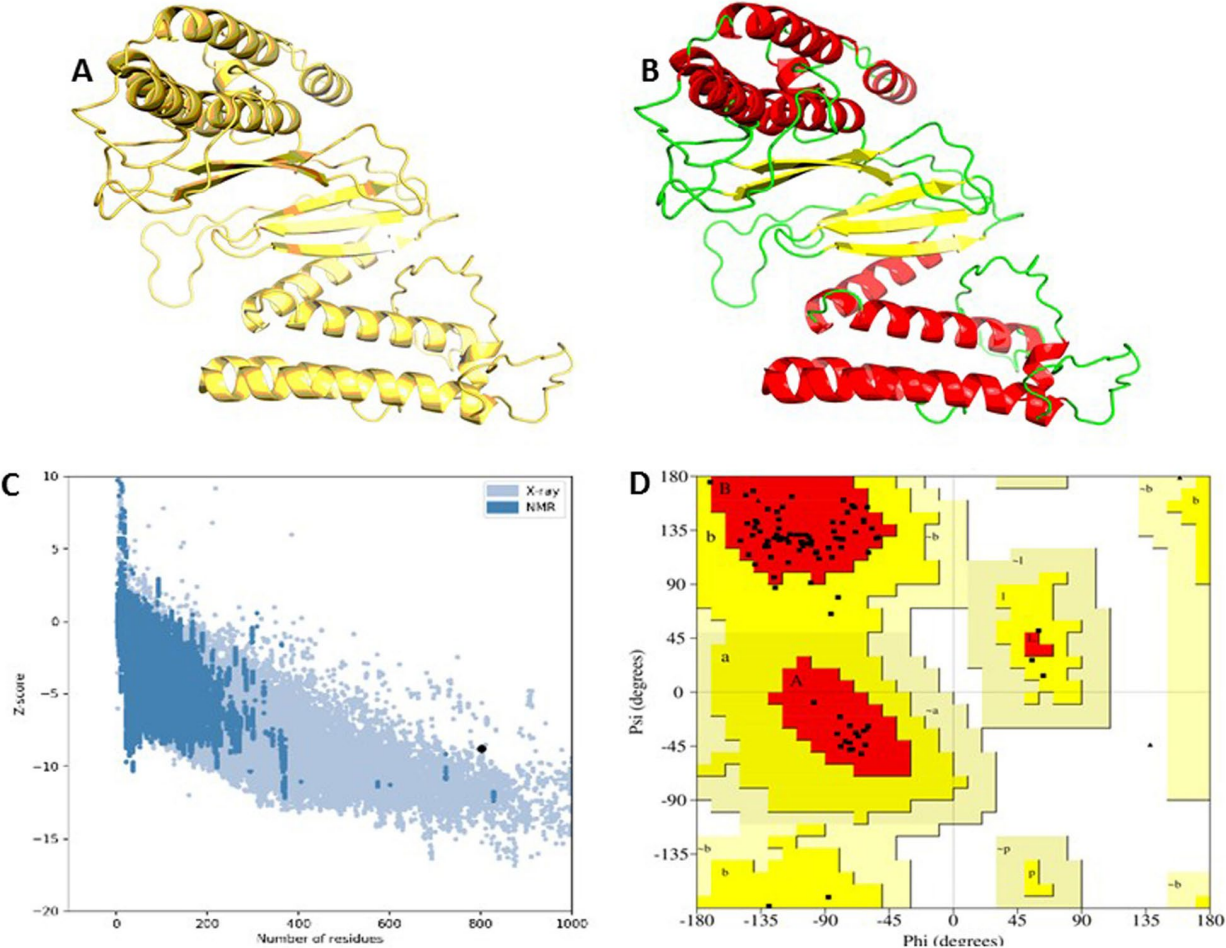
Validation of the final vaccine 3D models (**A**, **B**). PROSA 3D structure validation showing corresponding *Z*-score (− 8.77) (**C**). Ramachandran plot shows most favored (87.5%), allowed (12.5%), generously allowed (0.0%), and disallowed regions (0.0%) respectively (**D**)

The PROSA 3D server was used to predict the 3D structure of the multi-epitope vaccine sequence, affording ten predicted structures for a given query sequence. The model five was taken for further investigation in Fig. 3. The ERRAT, ProSA-web, and PROCHECK services were used to validate the structure, identifying and correcting any potential mistakes in the projected tertiary structure. The ERRAT server projected the overall quality of the vaccine 3D structure, with an estimated quality score of 95.0%. The *Z*-score was calculated to see if the input structure was within the range of similar-sized of natural proteins. Figure 4 shows that the computed *Z*-score for the input structure was − 8.77, indicating that it was outside the normal range for natural proteins of the same size. For Ramachandran analysis, the PROCHECK server computed 87.5% of the residues in the most favored areas, 12.5% in extra permitted regions, 0.0% in generously allowed regions, and 0.0% in forbidden region, with 0.0% residues in disallowed regions.

### Molecular docking

**Fig. 5:**
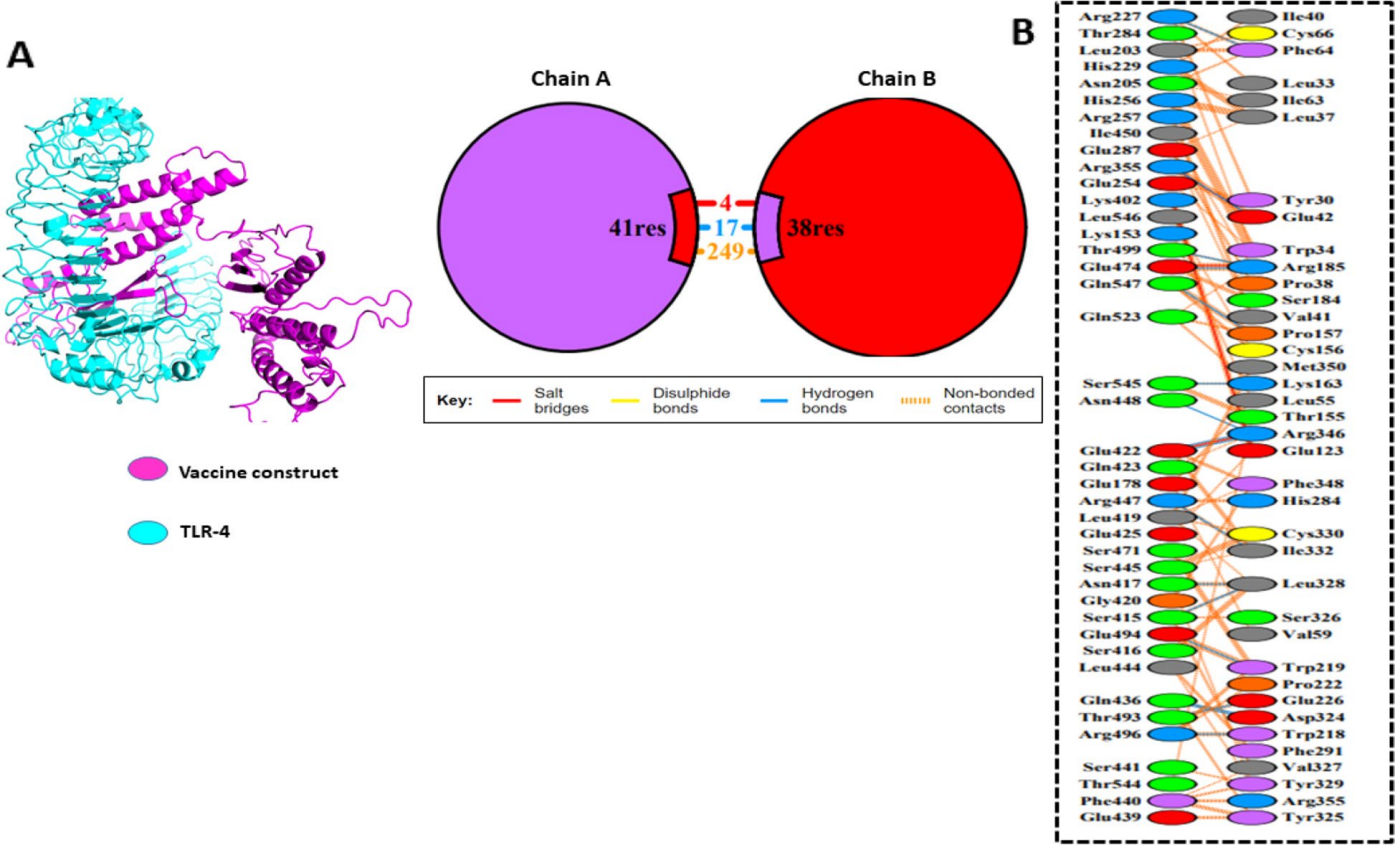
TLR-4 (PDB ID: 4G8A) vaccine docked complex. **A** The TLR-4 (receptor) is shown in light green, while the purple color represents the multi-epitope subunit vaccine. **B** Graphical representation of hydrogen bonds interaction between TLR-4 (ChainA) and vaccine complex(ChainB)

TLR-4 and the vaccine construct was docked using the Clustpro docking server to understand the interaction and to predict the final 3D model. A totals of ten models were created. Using the Pymol program, all ten docking models were visually examined and analyzed, Among the 10 model docking complex model 5 proved good docking result with total 17 H-bond interaction with the binding score − 290.5. For a graphical illustration of the residual interaction between the vaccine construct and TLR-4, the PDBsum online databases were used. To show hydrogen bonding between the vaccine construct complex and TLR-4, a graphical picture was generated. There were 17 hydrogen bond interactions between TLR-4 and the vaccine construct. Analysis of the vaccine-TLR complex involved the direct interaction of the residues which revealed that hydrogen bonds were formed between ARG355-GLU42, ARG227-PHE64, GLN547-PRO157, SER545-LYS163, GLU474-ARG185, THR499-ARG185, ARG496-TRP218, GLU494-TRP219, THR493-GLU226, GLN436-ASP324, GLN436-ASP324, SER415-LEU328, ASN417-LEU328, ARG447-ILE332, GLU422-ARG346, GLU422-ARG346, ASN448-ARG346 with distances of 2.82 Å, 2.81 Å, 2.85 Å, 2.70 Å, 2.70 Å, 2.73 Å, 2.67 Å, 2.65 Å, 2.89 Å, 2.86 Å, 2.96 Å, 3.13 Å, 2.72 Å, 2.86 Å, 2.63Å, 2.73 Å, 2.79 Å, and 2.93 Å, respectively Fig. 5 and Table 5.

**Table 5:** Represents the eigen value of all the 10 models

Cluspro 10 model	eigen value
Model-1	9.838369e−06
Model-2	10.159836e−05
Model-3	9.889836e−05
Model-4	9.988331e−04
Model-5	9.798369e−06
Model-6	10.542067e−05
Model-7	10.197361e−06
Model-8	9.874983e−06
Model-9	10.478327e−06
Model-10	9.987451e−06

### Molecular dynamics simulation

**Fig. 6:**
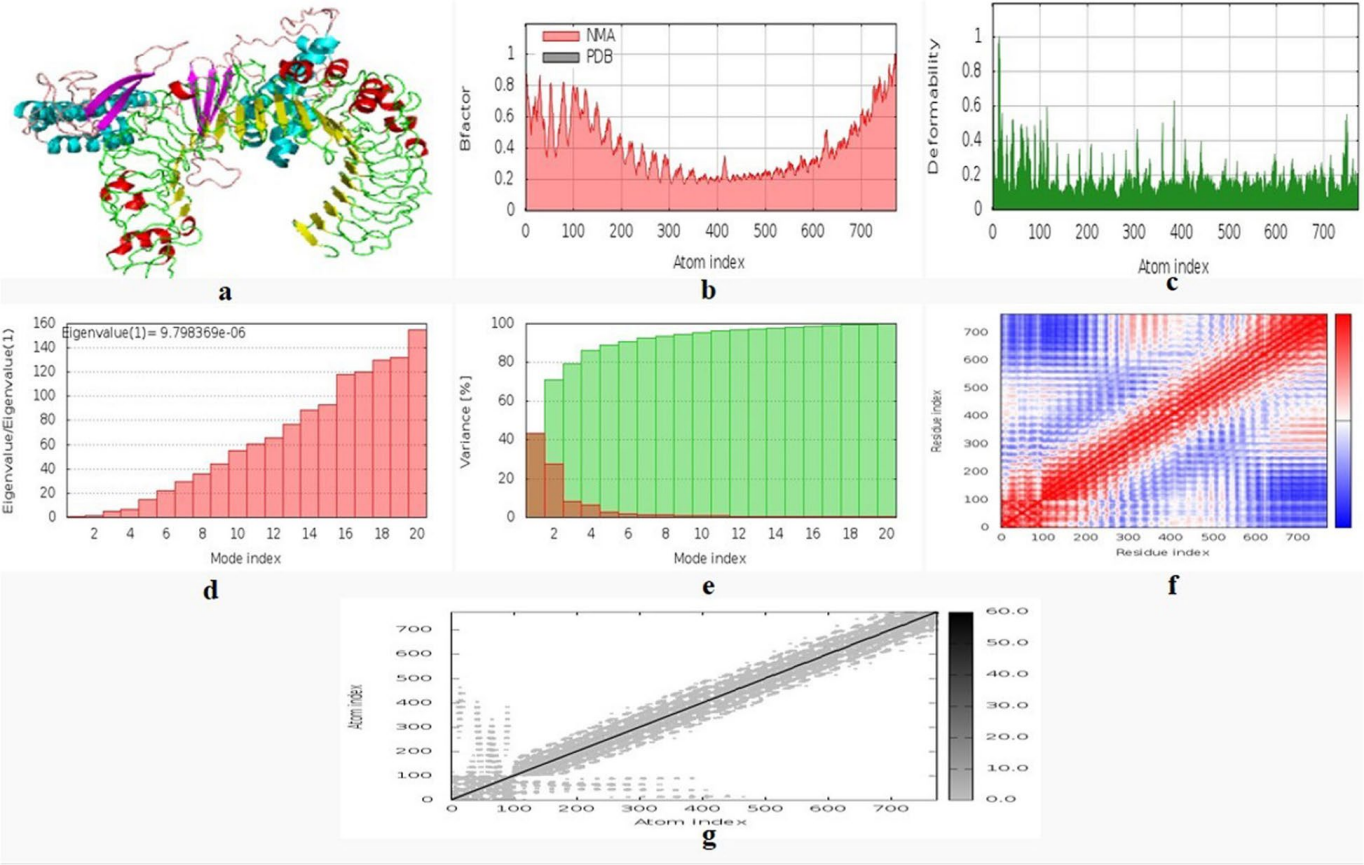
MD simulation of multi-epitope vaccine complexed with the TLR-4 **a** MNA mobility, **b** deformability, **c** B-factor, **d** eigen values, **e** variance, **f** co-variance map red (correlated) and white (uncorrelated) or blue (anti-correlated) movements, and **g** elastic network

iMODS performs an expository study of the structure by altering force field of the complex with regard to various time periods. The final model shows less deformation at each residue capacity level. The complex has an eigen value of 9.798369e−06. In heat maps, low RMSD and highly co-related regions indicated improved interactions between individual residues (Fig. 6). The figure gives a full description of the molecular dynamics simulation. The MNA mobility of the provided protein structure (b) component exhibits deformability, which reveals a low amount of deformation at all residues. (c) The B-factors and (d) the eigen values of 9.798369e−06 are indicated, and (e) shows the variance explained in both red and green hues. The co-variance and elastic network of the complex are also depicted in the other (f) and (g).

### Codon optimization

**Fig. 7:**
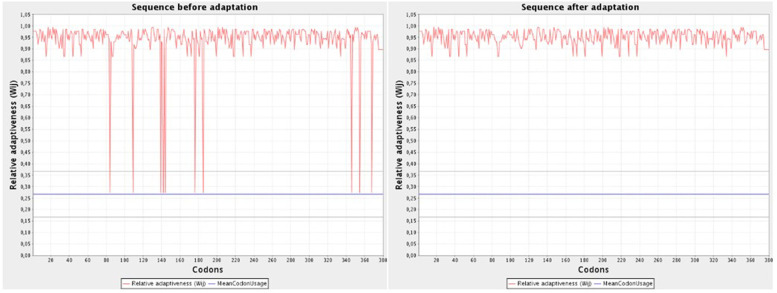
Codon optimization of design vaccine show sequence before adaptation and after adaptation

JCat, a Java codon adaptation tool, was used to optimize codons for maximal protein production. The optimized codon was 1140 nucleotides long and had a codon adaptation index (CAI) of 0.95, with a GC content of 63.42%. Because the GC content ranged between average of 35 and 60%, these figures imply steady vector expression in *E. coli.* (Fig. 7).

### Immune simulation

**Fig. 8:**
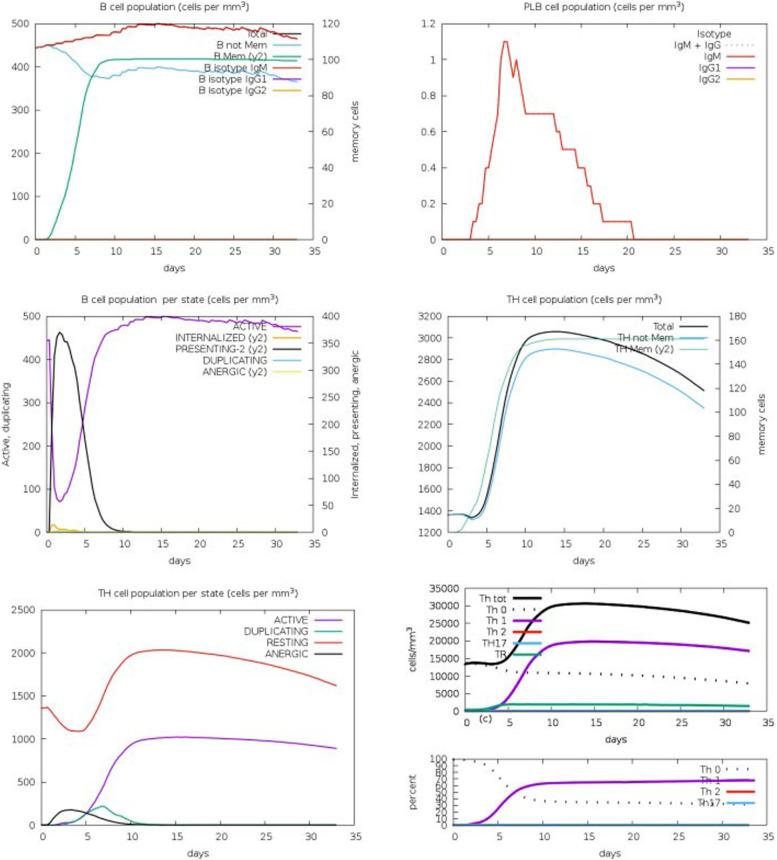
Vaccine immune simulation through C-ImmSim server shows production of antibodies, B cell and T cell population and cytokine production

We evaluated the vaccine's ability to trigger an effective immune response in practical use using the C-ImmSim webserver. After the primary reaction, the secondary and tertiary immune responses rose steadily. Antibodies (IgM, IgM+ IgG, and IgG1+IgG2) levels have risen dramatically. Figure 8 represents immune response showed a similar response to other immune responses in the human body. Results represent the production of IgG and IgM antibodies, high level of antibodies production and the IFN-γ score was also high as shown in Fig. 8. TH cell population indicated in Fig. 8.

## Discussion

The disease is causing global issues, and now is the moment to take preventative measures. Computational methods have become increasingly important in the design of multi-epitope-based vaccines. Multiple epitopes are highly recommended because of their cost-effectiveness, and higher efficiency. The study aims to design a vaccine against SARS-CoV-2*,* by using the immunoinformatics technique. The protein sequence was reported as the near-complete genome sequence of a SARS-CoV-2 Delta variant strain in Bangladesh, after which the antigenicity, Non-Allergenicity, and non-toxic properties of the protein were investigated. Mapping of epitopes was performed for further analysis. MHC-1 and MHC-2 were predicated. The selected epitopes are combined by the linker (EAAAK, CPGPG, and AAY). The stability and efficiency of the vaccine constructs were checked via an online server. Furthermore, clinical research on MHC targeting with human leukocyte antigen (HLA) is emerging [30]. Antigenicity prediction, flexibility, accessibility, hydrophobicity, and the antigenic tendency of polypeptide chains associated with epitopes position were all considered when developing a vaccine. By screening T cell epitopes with IC50 less than one hundred representing as highly active against alleles. MHC-I and MHC-II-linked epitopes were screened for analysis. VaxiJen v2.0, peptide ACC calculation was analyzed on basis of physiochemical. Peptides with more value than the threshold are considered antigens. Antigenic epitopes must be non-toxic to produce the optimal immune response. The ToxinPred tool was used to predict peptide toxicity. In vaccine designing, Allergenicity is the dominant obstacle [31]. For immune system stimulation, most vaccines create an allergic reaction. For allergic reaction prediction, The Aller Top v. 2.0 works [32]. This process is based on an auto cross-covariance (ACC) screened epitope with no Allergenicity. But according to WHO or FAO, for predicting Allergenicity, a sequence may be considered allergic if it contains 6 amino acid residues when compared with the database (known allergens amino acids) [33]. According to AllerTop v2.0, our epitopes did not accomplish the criteria thus it considered non-allergens. The epitopes with no toxicity and no Allergenicity make them immunoreactive peptides to be further processed [34]. Population coverage was also performed via IEBD so MHC molecules are polymorphic and exhibit thousands of alleles (HLA) [35]. Analysis of T cells with HLA were performed. MHC I and MHC II display high conservation in the world and also among Bangladeshi people. Epitopes of MHC-I and MHC-II of T cell and B cells epitopes were chosen for vaccine construction. By using suitable linkers B and T cells epitopes were linked for identifying multi-epitopes subunits vaccine construct. Bioinformatics analysis directed that our constructed protein sequence is non-toxic and non-allergic. However, the antigenic score was satisfactory if linked with adjuvant or not displayed through constructed vaccine chimera. The designed vaccine molecular weight was 50689.18 KDa, and the protein was further screened for solubility. Theoretical vaccine PI was 8.96. The value of vaccine instability was 42.46, which represents design vaccine is stable. Based on aliphatic index calculation, the constructed vaccine was considered thermostable. For vaccine designing, both secondary and tertiary structures are vital. Molecular docking was performed to predict protein-protein interaction. Docking was performed by PDBsum and PDBePISA representing a model of vaccine receptor complex showing the most promising interaction compared to reference one. Our result indicated less RMSD and fewer docking scores display stable interaction. In the receptor-binding pocket, the presence of van der Waals, strong electrostatic hydrophobic interaction, and hydrogen bonds make stable ligands [36]. To mimic the natural behavior, we performed an MD simulation. Deformability values, eigenvectors, B-factor, c-variance, complex MNA mobility, and complex elasticity in Fig. 6. During an immune response, stability and very less chance of deformations were observed because of the maximum eigen value. Through co-variance matrix analysis, immune stimulation of the designed construct was disclosed. Because of vaccine introduction in the body, a humoral response was expected to produce [37–40]. Through immune stimulation, an immune response was produced. As antigen exposure increases, immune response also increases. Memory B cells and T cells simulation are developed. After 1st injection, increased production of IL-2 was indicated. Codon was optimized according to host to get maximum expression results in GC was 63.42% and was CAI 0.95108334. It may prove a significant vaccine candidate if want to be experimentally performed in vitro and in vivo [39, 40].

## Conclusion

Using immunoinformatics methods a convenient method of vaccination was done against SARS-CoV-2 in Bangladeshi patients. This scientific endeavor starts with the NGS sequencing. Various immunoinformatics methods were utilized to create the vaccine protein. Online tools were utilized to build B cell and T cell epitope vaccines. CTL and HTL epitope were joined using adjuvant and linker. Then antigenicity and allergenicity were determined to ensure the vaccination is safe and stable. Molecular docking and MD modelling were used to test human-TLR-4 interaction and stability. Using immunoinformatics technologies ensures that the vaccination design is safe and stable.

## Data Availability

The sequences of SARS-CoV-2 genome from Bangladesh were submitted in the GISAID database accession no. EPI_ISL_803867. The datasets generated and analyzed during the current study are available from the corresponding author on reasonable request also. SRA number: BCSIR-NILMRC_355 SRA-SRP366148 (PRJNA818502)

## Abbreviations

MHC: Major histocompatibility complex
TLR: Toll-like receptor
NCBI: National Center for Biotechnology Information
IEDB: Immune Epitope Database
CAI: Codon Adaptation Index
GC: Guanine-cytosine
